# Strengthening resilience through an extended postnatal home visiting program in a multicultural suburb in Sweden: fathers striving for stability

**DOI:** 10.1186/s12889-019-6440-y

**Published:** 2019-01-22

**Authors:** Kirsi Tiitinen Mekhail, Lene Lindberg, Bo Burström, Anneli Marttila

**Affiliations:** 1 Department of Public Health Sciences (PHS) K9, Karolinska Institutet, 171 77 Stockholm, Sweden; 2 Center for Epidemiology and Community Medicine, Stockholm County Council, Stockholm, Sweden

**Keywords:** Fatherhood, Child health care, Extended postnatal home visiting program, Migrant, Parental support

## Abstract

**Background:**

To improve prerequisites for better health development among children growing up in multicultural suburbs in Stockholm County, where poorer health is displayed in several aspects including child health, early support was initiated for first-time parents in one of the suburbs. An extended postnatal home visiting program during the child’s first 15 months was offered to families with first-time mothers during 2013–2014 and consisted of six home visits by a child health care nurse and a parental advisor from social services. Almost all invited families (94%) participated in the program and the program evaluation. Fathers’ participation in two or more home visits within the program was 53%.

The aim of this study was to explore the experiences of fathers participating in the program, with respect to their role as a first-time parent from a resilience perspective.

**Methods:**

In-depth interviews were conducted with nine fathers. Constructivist grounded theory (GT) was applied in the analysis.

**Results:**

The fathers’ experiences formed the core category of the study, ‘striving for stability in living conditions’, as well as three categories: ‘everyday life conditions’, ‘adjustment to fatherhood in Sweden’ and ‘channels of support’. The fathers perceived that the home visiting program strengthened their parental confidence and increased their knowledge of societal services and local resources for their family.

**Conclusions:**

In terms of resilience, the extended postnatal home visiting program benefitted the interviewed migrant fathers on an individual level by meeting part of their need for support regarding knowledge and parental confidence; on a structural level the program helped fathers gain information about available societal services and resources in their local area.

**Trial registration:**

The study was retrospectively registered (11 August 2016) in the ISRCTN registry (ISRCTN11832097 DOI: 10.1186/ISRCTN11832097).

**Electronic supplementary material:**

The online version of this article (10.1186/s12889-019-6440-y) contains supplementary material, which is available to authorized users.

## Background

The national goal of public health in Sweden is to create societal conditions for good health on equal terms for the entire population [1]. Swedish Child Health Care (CHC) services work to promote children’s health, to prevent illness and to initiate actions when problems are discovered in health, development or upbringing [2]. Parental support is already well established in Sweden, and through local Child Health Care Centers (CHCC), CHC services essentially reach all families with children in Sweden [3].

There are substantial health inequalities within Stockholm County [4]. The multicultural city district of Rinkeby-Kista, with more than 90 % of the population having a migrant background (born outside of Sweden or in Sweden with two foreign-born parents) [5], is one of the disadvantaged districts, displaying poorer health in several aspects; children’s health in the area is affected [4].

To improve prerequisites for better health development among children, early support was initiated for first-time parents in the Rinkeby-Kista district by CHC services and social services [6, 7]. The extended postnatal home visiting program in Rinkeby includes six home visits during the child’s first 15 months, where new parents are visited by two experts: one CHC nurse and one parental advisor. By contrast, the ordinary CHC program in Sweden only includes one home visit by one child health nurse. The CHC nurses provide health care support, while the parental advisors, who are trained social workers, provide psychosocial support, especially regarding family relations and interactions. The program was offered to all first-time families, who chose to register their child at Rinkeby CHCC from the 1st of September 2013 to the 31st of December 2014 [6, 7]. A first-time family is defined as the mothers’ first child. Both parents were encouraged to participate in the program by the CHC nurses and parental advisors during the whole home visiting program. Ninety-four percent of the included families wanted to participate in the home visiting program and signed a consent from before participating in the program evaluation [6, 7]. Around 79% of the fathers attended at least one extended home visit [8]. There is some estimation from the literature that up to one-half of fathers participate in home visiting programs to some extent when fathers participation is a focus in the programs [9].

The extended home visiting program of this study follows the guidelines of the Swedish CHC program [2] and the visits are integrated in the universal CHC center-based services, including themes about development, safety, nutrition, interaction, parenthood, social network and support [6, 7]. Parents are supported in their parental roles, and their questions are discussed from a perspective emphasizing resilience, promoting health, and supporting and encouraging a positive parent-child relation [6, 7]. A manual has been created for the extended home visiting program by CHC nurses and parental advisors in a parallel process within the intervention [10].

The published study protocol of the extended postnatal home visiting program describes housing, as well as the financial situation of the participating families. Preliminary findings show parental insecurity after child birth, and that home visits seem to meet this need and are appreciated by the families, as well as by the professionals that are engaged in the program [6].

The final evaluation report of the home visiting program (in Swedish), based on questionnaire-based interviews and analyses of medical records, reports increased confidence in the new parental role, increased knowledge of the Swedish society and support for parenthood, as well as increased trust in the Swedish health care system through close relationships with home visitors. Increased coverage of MRR immunization is observed in the study site, as well as at least temporarily decreased utilization of emergency care [11].

The analysis of the content of the meetings between families and professionals during the home visits based on the CHC nurses’ documentations reveal that the home visits within the extended home visiting program covers three main categories of content related to i) the health, care and development of the child, ii) the strengthening of roles and relations within the new family unit, iii) and the influence and support located in the broader external context around the family [8].

Fathers’ experiences and their perspectives on home visiting are the focus of this study, as it is known that fathers play an important role in children’s social, emotional and cognitive development [12] and their lower attendance in other studied home visiting programs means that we know less about their experiences.

Migrant fathers’ descriptions of fatherhood in earlier studies include both stress, joy and pride, as well as a feeling of being overwhelmed [13]. Entering fatherhood has been described further by migrant fathers as a life-changing experience that includes their responsibility to raise the child [14]. In earlier research migrant fathers describe their strong desire to be family providers [13, 15–19], which may be challenging in the new country when the work does not always correspond to their level of education or skills [13, 16, 17], employment is insecure, jobs are low-paid [16, 17] and racism is present at the workplaces [19]. Some migrant fathers express that unemployment can contribute to feelings of isolation; a lack of productivity, a negative effect on finances and hinder integration [14]. Housing issues, together with other consequences and stressors of migration, can challenge migrant parents [20]. Earlier studies have also determined that misleading information, language difficulties [20], cultural differences, lack of social resources and financial stress [21] may hinder migrants’ participation in society. Lacking a social network that provides practical help and knowledge can further challenge parenthood generally [22–24], as can relationship issues, health and finances [24].

In earlier studies, migrant fathers describe the time spent with their children as important [14, 16], although lack of time to do so was mainly caused by work [15, 16, 19]. Migration sometimes offers fathers more time with their family compared with life in their home countries [13]. Activities at home and outside, and participating in their children’s everyday routines are mentioned among the activities of paternal involvement [13, 15, 16]. Communication with children can be important [19], and is also cited as a major factor for successful parenting by some migrant parents [20].

The family’s well-being, health and access to health care are important for migrant fathers [16], and a good education for the children [13, 16, 17, 21], seen as guaranteeing a better life for the next generation, is sometimes the primary factor inspiring migration [16]. Extended possibilities in the new country for the life of children and families, including parks and playgrounds as well as safety, security and peace are mentioned [13, 16, 21]. Migration can give financial benefits [17], and well-organized health care systems are appreciated [13, 14].

Migrant parents often note the lack of original social networks in their new countries [13, 16, 17, 20, 21]. Parental tasks and responsibility are described as being shared more collectively with relatives in home countries [13, 20, 21]. The lack of social networks may cause isolation, and parenthood may be perceived as a difficult task in the new country [13, 16, 20, 21]. Retained contacts with families in the home country can provide consultation and support [16]. The paternal role in child care may expand due to a reduced network [17, 21], but adjustment to the new role can also be stressful [22].

As social networks may be lacking for migrant women, fathers are described as participating within maternity care and at childbirth in their new countries [14, 18]. Involvement within maternity and child care is mentioned by some migrant fathers to challenge traditional values and perceptions about masculinity [18]. Middle Eastern women in Sweden note that men do not always succeed in replacing the support provided by female networks during the stressful postnatal period [25]. Migrant men’s participation in household duties is sometimes expressed as a change compared with their corresponding role in the home country [16].

A previous study in Sweden report that migrant parents might feel vulnerable and concerned about being misjudged when visiting CHCC [26]. Parents may feel confident, hesitant or unwilling to continue contact with CHCC, depending on how the interaction with the nurse develops [26]. Practical, individual tailored advice within maternal or child health care is described as important and used in combination with advice from their own social networks [14].

Non-European migrant parents express gratitude when comparing Swedish CHCC with health care provisions in their home countries [27]. Home visiting is appreciated, and parents are mainly content with the involvement, oral and written information and parental advice provided by CHC nurse and an easy access to CHCC [27].

Postnatal home visiting programs have historically focused on mothers [28, 29]. The outreach provided by parental prevention programs is argued for in order to increase fathers’ engagement with children [28, 30, 31]. Increased paternal involvement is reported to have positive child outcomes [28]. Among the challenges in engaging low-income fathers in home visiting programs in the USA are recruitment into programs, keeping fathers engaged, schedule-related issues, staff resistance, maternal gate keeping, and fathers’ perceptions of home visiting and meeting the needs of particular populations, including those of nonresident, migrant, and teen fathers [32].

In looking at different national home visiting programs in the USA, research shows little evidence, that paternal involvement into home visiting programs may increase their involvement and improve children’s outcomes [33]. Programs such as Early Head Start and Healthy Families America promote fathers’ involvement and engagement [33]. Nurse-Family Partnership targets mainly mothers but also welcomes fathers’ participation [33].

One example of more recent studies promoting fathers’ participation in early home visiting services for vulnerable families is The National Healthy Start Association’s ‘Where Dads Matter’ initiative in Midwestern metropolitan areas in the USA. Findings from a pilot study including fathers with different ethnic backgrounds (*n* = 12) indicate positive trends associated with the quality of the mother-father relationship, perceived stress reported by both parents, fathers’ involvement with the child, maltreatment indicators, and fathers’ verbalizations toward the infant [30].

Results from an evidence-based home visiting program Healthy Families New York, (HFNY), which targets expectant and new parents in socioeconomically disadvantaged families at elevated risk for child maltreatment and other adverse outcomes, shows that when fathers participated in home visiting, families were more than four times as likely to be retained in the program. Families (*n* = 3341) had different ethnic backgrounds. Fathers who are engaged in the program are more likely to live at home with the child and to remain emotionally involved at 6 months follow –ups, supporting the need of policies and practices to encourage participation of fathers in high-risk families in home visiting services [9].

The Family Nurse Partnership’s (FNP) home visiting program in England, which focuses on young fathers, including those with different ethnic backgrounds, is another example of home visiting programs involving some migrant fathers [22]. The FNP program focuses on strengths as well as on areas in need of development, and has showed results in improved parental skills and decreased anxiety about child care [22]. Fathers’ involvement increases over time, and the program helps with relationship issues, and the development of practical skills, leading to increased parental self-confidence [22].

More examples of the successful use of targeted home visiting programs to risk families are found in the USA [34] and Finland [35], showing long-term effects including fewer psychiatric symptoms for children as young adults compared with control families [35] and improved academic adjustment to elementary school [34].

Taken together, there appears to be the will, but also challenges to involve fathers in postnatal home visiting programs, and there is limited knowledge about home visiting programs involving fathers, especially those with a migrant background.

This study focused on the experience of fathers participating in a home visiting program. Resilience was chosen as the theoretical perspective of this study. Resilience as a concept has different definitions in the literature but is defined in this study as “the ability to successfully adapt to stressors, maintaining psychological well**-**being in the face of adversity” [36]. The extended postnatal home visiting program in Rinkeby has a resilience perspective, operationalized in terms of strengthening parental self-efficacy, trust, and access to local community and health care services [7].

### Aim

The aim of the study was to gain in-depth knowledge of the parental experiences and needs of fathers, who took part in an extended home visiting program in a multicultural suburb in Stockholm, Sweden.

## Methods

The study used a qualitative design. Constructivist grounded theory (GT), following Charmaz [37], was used to analyze in-depth interviews with fathers. According to Charmaz’s guidelines, rich data collection is followed by initial line-by-line coding, focused coding, axial coding and theoretical coding [37].

### Setting

Inclusion criteria for the study were that fathers belonged to the families participating in the extended home visiting program, which was offered at the study site from September 2013 to December 2014, and participated in more than one of the home visits, as all families in Sweden that have a child are offered one home visit by a CHC nurse [2]. The study aimed to explore experiences of fathers who participated in the extended postnatal home visiting program by seeing a CHC nurse and parental advisor and attended at least two home visits.

**Fig. 1:**
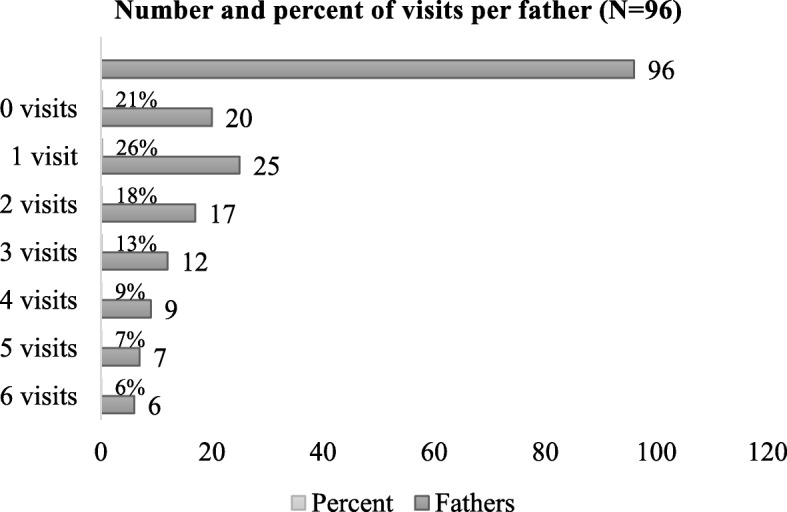
Number and percent of visits per father in the home visiting program

For the recruitment of participants, contact information was provided to the first author by two CHC nurses who worked with the intervention. In total 119 families were asked to participate in the home visiting intervention; 17 of the families dropped out for different reasons, and one third of the mothers in the included families were not living with the child’s father [7]. In the participating families, 96 fathers were known to have had any contact with the mother and the child. Overall 79% (*n* = 76) of fathers attended at least one home visit [8], and 53% (*n* = 51) attended two or more home visits and were eligible for the interview study (Fig. 1). When recruiting among these 51 potential participants, the goal was to have a heterogeneous sample of fathers regarding age, life situations and experiences, an approach known as maximum variation sample [38]. However, the CHC nurses became the key persons for the recruitment, as they provided the first author with a list of contact details of 33 fathers that were available for the interview study.

Fourteen of the contacted fathers accepted to be interviewed. However, five cancelled the interview at the last minute. For ten others fathers, who did not participate, lack of time or interest, family situation or longer stays abroad, were some of the reasons to not participate. Nine fathers could not be reached by using the provided contact information, despite multiple calls and text messages. Therefore, a total of nine fathers participated in in-depth interviews by the first author, who is a CHC nurse in the local district, with knowledge about the study population, but with no previous relationship to the participants. One of the nine participating fathers had attended all six home visits, four fathers attended four home visits, one father attended three home visits and three fathers had attended two home visits. Interviews were conducted between October 2015 and November 2016, when informants’ children were 16 months or older.

Eight of the nine participants were first-time fathers, aged 25–45 years, having non-Nordic origin. One father had grown up in Sweden, but had two non-Nordic origin parents. Other interviewees had grown up in non-European countries and had lived in Sweden between 6 and 22 years (Table 1). A more complete description of the origin of the families is reported in a Swedish report [7]. All of the interviewees had at least a high school education, and seven of them were employed at the time of their interview, while two were unemployed (Table 1).

**Table 1:** Demographic characteristics of interviewed fathers

	*N* = 9
Paternal age - Mean (SD)	35.2 (25–45)
Paternal origin (N)	
Europe	1
Middle East	3
Africa	4
Asia	1
Residence in Sweden (years) – Mean (SD)	8.9 (6–22)
Years of education (years)	
< 12	0
≥ 12	9
Employment	
Employed	7
Unemployed	2

### Data collection

The participants were provided written and oral information about the aim of the study. Oral informed consent was obtained before interviews, as all the families had agreed earlier with written consent to participate in the evaluation of the intervention. Three of the interviews were conducted at the local CHCC and six at the homes of the participants, according to their choice. During the interviews conducted in homes, informants’ wives and children were present.

In-depth interviews were conducted to gain an understanding of the fathers’ perspective and to explore their experiences [37]. An interview guide was used including the following themes: health and well-being, the child, the extended home visiting program and future (Additional file 1). The first pilot interview was included in the study, as it was informative for the study and the interview guide was perceived as well-functioning. After five interviews, the ongoing initial categorization appeared to be consistent with the new interviews. Four further interviews were conducted to reach saturation. However, new information was not gained from the further interviews, as the same aspects and phenomena were repeated by the fathers.

The participants could choose if they wanted to be interviewed in Swedish, English or to have an interpreter speaking their primary language. Six interviews were conducted in Swedish, two in English and an Arabic-speaking interpreter was present at one of the interviews. Interviews were audio recorded. Memo writing was used to help the first author in developing thoughts and ideas during the research process [37].

Interviews lasted 20–45 min. After every recorded interview, the interview was transcribed verbatim. On two occasions, two interviews were conducted before the transcription and coding of the previous one was finished. At the end of each interview, the informant was asked if he had any further questions, comments or corrections about what was said during the interview.

Evaluation of the intervention, including in-depth interviews, received ethical approval from the Stockholm Regional Ethical Review Board (Dnr 2013/877–31/1).

### Analysis

**Fig. 2:**
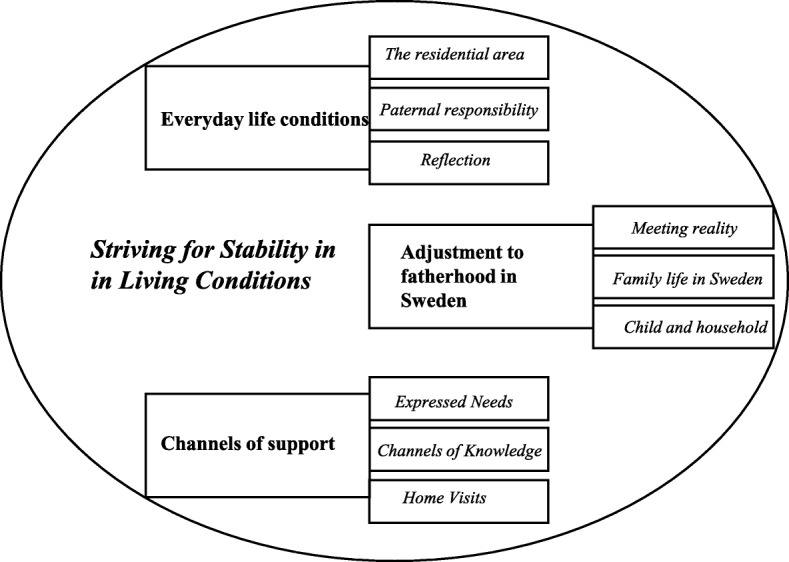
The model of categorizing

The analysis was based on Charmaz’s [37] guidelines for GT, leading inductively to the study results. The computer program Open Code 4.03 was used for open, line-by-line coding, to name each piece of data with a label that simultaneously categorized, summarized and counted each part of the collected data. Text fragments, words, lines, segments and terms/expressions of participants were studied and seen as in vivo, living codes. Focused coding [37] followed initial coding and included; organizing codes into categories forming a code list. The most descriptive initial codes related to the study’s aim were chosen and tested with more comprehensive data. During the process of data analysis, data segments were compared with each other. Focused coding, that is, forming code lists with fewer written codes, was used to create more theoretical headlines. Axial coding [37] led to three more theoretical categories in the data, based on more theoretical headlines. The Core-Category of the study was formed to cover all the codes and categories that were included in the data analysis [37]. Results were described in the model of categorizing (Fig. 2) and were discussed by the research team.

To present quotes in the results section, each participant was assigned a letter (A-I). Quotes highlight the current code/category and illustrate the topics, showing the expressions of the interviewees, based on the interview data. Minor grammatical corrections were made in the interview quotes to assist the reader.

## Results

Based on the nine qualitative in-depth interviews, the findings of the study present analytical categories constructed from the interviews with fathers. *Striving for stability in living conditions* was the identified core category, describing the experiences of becoming a father and the paternal needs for support in parenthood. Three categories were constructed: *everyday life conditions, adjustment to fatherhood in Sweden* and *channels of support.* Categories are followed by subcategories (Fig. 2). The presented three categories are aiming to be specific, but under the subcategories ‘parental responsibility’, ‘meeting reality’ and ‘family life in Sweden’, the reader can discover some overlapping, as all these three subcategories are describing fatherhood.

### Everyday life conditions

When expressing the experiences of their everyday life, fathers described *the residential area*, *paternal responsibility* and used *reflection* to compare life conditions in Sweden with their countries of origin.

The residential area offered easy access to services needed in everyday life, playgrounds for children and reasonable housing rents in the capital area. The neighborhood’s problems with drugs, criminality, motorcycles being driven on sidewalks, and parents neglecting their parental responsibility were mentioned by interviewed fathers.

Fathers distanced themselves from the criminals; they could feel at home in the area, but they wished for a safer environment for their children to grow up in and wanted to move.

*-You cannot get involved with the criminals... you just need to... do your shopping, keep on living, pass by them... and not at all hang out in the center…* (Father G)

The family was a priority for the interviewees, who noted a strong feeling of responsibility, a wish to provide for the family and a desire to create stability. Some fathers had stable work; those unemployed were active jobseekers. Both unemployment and hard work could cause stress.

*... I work a lot, too much... I have two different jobs... at the moment I am taking leave from one of them… to be able to keep on… I am standing up all the time, sometimes more than 12-13 hours...* (Father D)

The housing situation was a challenge, short-term contracts were common and the housing market in Stockholm was seen as catastrophic. A long wait for housing could result in short rental contracts, which meant that families kept on moving. A wish to find more suitable housing in a better neighborhood was generally expressed.

Regarding their feelings about the importance of education, fathers considered preschool to be important as the first step of learning outside the home; preschool helped the children to learn Swedish, become social, develop fixed routines, and have playmates.

*... The mother didn’t think to send our child to the preschool, because she is still so young. But I told her “no”, here in Sweden, they are going to learn… the mother cannot teach her at home, not like in the preschool… so the mother said “OK”.* (Father D)

Both their own health and the health of the whole family were important for these fathers. Health was linked to experiences of being an active father and meeting the family’s needs. Interviewed fathers described their health generally as good, and some of them experienced even better health after becoming a father. Tiredness was caused by lack of sleep; stress was caused by work and various tasks at home, and was increased by housing and finances.

Fathers compared Sweden with their countries of origin, where problems such as war and financial struggles could be a reality. Sweden was considered to be a better and safer country with a functioning society, education and work, offering the child more possibilities. Child benefit in Sweden was mentioned as positive, and concerns for the future were lessened. Social networks were important but less available in Sweden; the lack of time in everyday life was observed to affect relations.

*...even if you have the family in Sweden, they do not have time to come and visit... and even if they come... they come two times a year... it is not that much.* (Father B)

### Adjustment to fatherhood in Sweden

Becoming a father was described as *meeting reality*. Fathers compared *family life in Sweden* with how it looked in their home countries and described their involvement with their *child and household*. The postnatal time at home was filled with joy and difficulties, as the parental role meant an enormous change for fathers. Holding the newborn baby was wonderful; the feelings were unimaginable and indescribable:

*... There are different feelings, you cannot explain what you are feeling exactly, but it feels very good...* (Father C)

To care for the newborn baby could be difficult for both parents, as they often knew very little about children. The reality was different from written information. Over time it became easier to change diapers and clothes, to feed and bathe the child. Visiting grandparents could be helpful during the first period:

*…my parents were here in Sweden for a while so they helped us… I think we managed; we coped with that… so I do not see any worries…* (Father I)

Breastfeeding was seen as a necessity by fathers, a natural way of feeding and calming down the newborn baby. Problems in breastfeeding could create stress for both parents. The early phases of parenthood also meant tiredness; lack of sleep declined as the child grew older.

The child demanded much patience from parents and provided a chance to develop responsibility.

*-The feeling of responsibility, yes, it is very hard for me... I have never needed to take responsibility for others. It was never my thing; if someone was sick, I wanted go from there... so I was afraid of that... but now there is a big difference with the child, a huge difference…* (Father C)

The early postnatal period at home could be hard, and fathers thought that this experience was the worst for first-time parents. Becoming a parent was seen as a process of education, the possible second child would be easier.

Fathers expressed gratitude for the support and help that Swedish society offered to parents; however, the lack of extended family meant more responsibility for fathers and mothers. Social networks, including relatives and friends, were important for the fathers, contributing to their well-being and decreasing loneliness. Some families had their extended family living close by, others far way:

*-We do not have relatives here...Skype and Viber... that means some hours every evening, one and half hours… it is important, especially with both grandmothers and… they want to see her (the child), she is the first grandchild in the whole family… everyone wants to see her all the time… they want to hear and see how she is growing and… it is exciting.* (Father G)

Fathers expressed a wish for practical support from their relatives in everyday life, for example, by leaving and picking up children from preschool, offering babysitting and helping with other such tasks.

None of the participants described having help in everyday life. The mother and father alone took care of the child and their practical life. Although some fathers described emotional support from family in their home countries, the help and support of the original social network was missed.

*... Especially for my wife, she could get more help, knowledge from her mother or from my mother and people around us...* (Father E)

Fathers expressed that having a child could improve their quality of life and being a father provided them with a different kind of joy. To have a child was also described as one of the greatest moments in their life and despite challenges, fathers did not express any major difficulties. The continuous development was described as one of the nicest things in fatherhood. Fathers described their children positively as they gave life at home, sought fathers’ attention and contact, and were playing together.

Based on interviews with fathers, the father-child interaction changed as the child grew up. Initially, the practical care for the child was the most important. Fathers learned to read the child’s signals, soothing with hugs, kisses and later by distraction. The child’s increasing ability to communicate helped fathers; the child could listen and understand more. Setting limits for a crying child could be hard but the child’s own will power was seen as part of normal development.

Participants mentioned active communication with their children and different activities such as watching TV or a film together, reading books, talking with the child, playing at home and spending time outdoors, especially in the summer. Tiredness after work did not hinder fathers from playing outdoors in the evenings.

The fathers said they were involved in household duties, which was not always the case in their home countries; both parents did their best for the child and helped each other. During the postnatal period, both parents took care of the child. Few changes in the marital relationship were mentioned; time was spent with each other even if the child needed total attention most of the time. However, there was a change.

*-It is a huge jump; it is not something bad but something new. You cannot say that it is exactly bad. We were arguing a lot as we were tired and so... it is quite common, I think, something that everyone is passing through...* (Father C)

Other descriptions noted that two became three and the relationship was unchanged since the birth of the child.

### Channels of support

Interviewees *expressed needs* for support in parenthood, and they seemed to actively look for the *channels of knowledge* and appreciated the postnatal *home visits* they received from nurses and parental advisors.

Fathers expressed needs for advice and support, especially as first-time parents who knew very little about children. Support was needed both regarding theoretical knowledge and practical skills.

*...you can know it in your head, but in the reality, it is difficult... I see other parents, my friends, but... when you are the parent, for the first time, it is not easy...* (Father F)

Making more information and knowledge related to child care available in languages other than Swedish, were suggested as possible offerings that could be provided by CHCC and the society.

In addition to the CHCC and home visiting program, fathers mentioned several channels of knowledge. Family, relatives and friends were sources for practical advice and parental skills. Family members who were far away were consulted through phone, Skype and Viber. Relatives working within health care became important:

*... I wished sometimes in the beginning... that I had my sister (who is midwife) here...she could help.... she (my wife) had it hard in the beginning…* (Father G)

The Swedish health care advisory call number (1177), maternity ward and Internet added some information of baby care. The different channels of knowledge could be confusing. To get assured about the acquired information, the reliability of advice was double-check with other sources, among them Internet. Education was thought to reduce confusion concerning conflicting advice.

*…the confusion is coming when you don’t have education… when you have education, no confusion…* (Father H)

Fathers listened to and compared different advice and made their own conclusions. Health care professionals were consulted to reduce anxiety. Sometimes the emergency ward was visited.

*We like the hospital, so we go there quite often... it could be whatever... the first child, we were afraid…* (Father C)

Fathers said in interviews that the waiting time for seeing a doctor at the emergency department could be long, which made them delay and see if that care was truly necessary.

Beside the good care, CHCC offered information and practical advice on topics such as food and sleep. The CHC nurse’s advice was often beneficial, and fathers were satisfied and appreciated the easy access; they kept in contact with the CHC nurse as the child got older.

Fathers described the extended postnatal home visiting program positively. The fathers expressed the number of home visits as good for the family; even more visits were suggested by one father who attended all the six visits. Participants did not think that home visits could be replaced by appointments at the CHCC, as the time for discussing parents’ questions at the CHCC was described to be limited. The themes covered were related to the child’s different developmental phases, giving parents a chance to get prepared for the following developmental stages. Several home visits to first-time parents were recommended:

*I think it is a must, it is very good for the parents... many times they do not know anything about how to take care of a child... with the second child... you have got little experience from the first child…* (Father A)

Home visits helped parents to gain confidence, and the CHC nurse and the parental advisor were perceived as caring.

Some fathers explained that the parental advisors focused on parental roles, gave information about the society such as open preschool and asked important questions about the feelings of the fathers; other participants could not specify the topics. Advice about behaviors, bringing up the child and general advice in parenthood were mentioned:

*... We got very good advice, especially when he is growing up, behaviors and so, general questions that we got answered, we got it quite a lot... good advice how you can do certain things...* (Father C)

Two of the interviewed fathers perceived that during the home visits parents were asked quite a number of questions by the parental advisors and the CHC nurses. These fathers did their best to answer the questions addressed to them.

## Discussion

This study aimed to gain in-depth knowledge of the parental experiences and needs of migrant fathers who participated in an extended postnatal home visiting program, from a resilience perspective. A core category and three linked categories presented in this study are based on nine qualitative in-depth interviews with fathers.

The study findings showed that the program benefitted the interviewed migrant fathers by meeting part of their needs for support on an individual level, both regarding knowledge and parental confidence. Further the home visits strengthened the fathers on a structural level by giving them information about available societal services and resources in their local area. Even if the participating fathers had been living in Sweden for at least six years at the time of the interviews, they still were in need of more information of societal services in their local area, which may indicate that fathers who have lived for a shorter time in Sweden, may have an even greater need of information about different societal services.

The study suggests that the unique combination of two professions in collaboration was making the extended home visiting program successful and appreciated by the fathers, as the nurses contributed with health care support and the parental advisors with psychosocial support. In the interviews, the fathers did not differentiate between the two professions, but appreciated the received support.

A main finding was the participants’ striving for stability in the family’s living conditions in the multicultural neighborhood. Similar experiences with the extended home visiting program were also expressed by mothers interviewed regarding the program [11].

### Everyday life and resilience

Resilience was not specifically mentioned in the study findings, but could be identified out of the descriptions provided by the interviewed fathers, as the inherent strength that made them keep on investing time and effort in striving for stability for their families in everyday life, despite various challenges, which is also described in previous studies, such as work-related issues, socio-economic conditions [13, 14, 16–19], housing issues [14, 20] finances, health, relationships [23] and lack of parental skills and knowledge [22–24].

### Adjustment to fatherhood

The lack of original social networks is, according to some previous studies, common among migrant parents and can complicate the parental task [13, 16, 17, 20, 21]. The interviewed fathers expressed their adaptation to the new reality of fatherhood and their involvement in the family, given that the natural social networks were smaller in Sweden compared with home countries. Ways of adaptation included retaining contact with relatives back home [16], having social contacts in the neighborhood and increased paternal involvement in the child’s issues which, according to earlier findings can bring fathers closer to their children [16, 21]. As in other studies, we found that migration may offer a new dimension in fatherhood [16], more involvement in child care [19] and more time spent with family. The relatively high attendance of the fathers in the studied extended home visiting program (Fig. 1), indicated their willingness to get involved with their children, and their descriptions in the interviews showed their ability to handle their new life situation as fathers.

Mixed feelings when holding the baby, from wonderful to helpless and inexperienced, were described by the interviewees in line with earlier findings [13]. Practical parenting skills [22], initially lacking, were developed over time by the interviewees through practical care of the baby. Communication [19, 20] and time spent with the child [13, 15] are noted as important for migrant fathers, but also challenging because of the lack of time [15, 16, 19]; such feelings were also expressed in this study. As this study focused on families with their first child, future studies should also investigate paternal experiences of migrant fathers who have several children.

### Channels of support

The interviewed fathers seemed to find channels of support for their expressed parental needs that could strengthen their ability to adapt (resilience) in the new life situation. The fathers could see available resources in Swedish society as visible benefits of migration in line with earlier studies [13, 16, 21]. Life was described as better and safer with the well-organized society in Sweden; improved possibilities for the children’s education and health care were appreciated by the interviewed fathers, as also reported in earlier studies [13, 14, 16, 17, 21].

Social networks and consultation with professional experts help migrant fathers gain knowledge and practical skills [14, 22]. In addition to CHCC, the Swedish health care advisory phone number (1177), the Internet and social networks were used by the interviewees of this study for knowledge on practical parental matters.

CHC nurses were experienced positively by interviewees. The importance of receiving advice and parental guidance through the CHCC was expressed; access to CHC nurses, when needed, was appreciated as seen in earlier studies [27]. For interviewed fathers the CHCC meant good care, medical check-ups, information, availability and much practical advice.

### The extended home visiting program

Fathers were satisfied with the number and content of the home visits. Home visits delivered information about Swedish society and benefitted fathers on the structural level, as they learned about available resources for parents and children in the local area. This can be interpreted as an indication of increased health literacy (HL).Participation of the parental advisors together with the CHC nurse during the home visits was appreciated by fathers. Parental advisors were described to offer ideas about parental roles and information about society and expressed care by asking about the feelings of the father and offering advice about the child’s behaviors and up-bringing.

The difficulties in recruiting fathers for the current interview study and the absence of them at some of the home visits during the intervention showed that not all the fathers participated or had the possibility of attending CHCC. Challenges in involving fathers within home visiting programs have been reported in earlier studies [32]. McGinnis et al. (2018) describe that up to one-half of fathers participate in the home visiting programs to some extent when their participation is focused in the programs. The participation of fathers at least once in the presented extended home visiting program was 79%, which is relatively high [8]. However, 53% of the fathers participated two or more visits of the program, which is similar to earlier findings [9]. The program of this study was targeting both parents and encouraging both mothers and fathers to participate, showing that it is possible to reach even migrant fathers through a parental home visiting intervention. An earlier study conducted in the Netherlands showed that both parents are more often involved with home visits compared with visits at the clinic [39].

The extended home visiting program could be seen as an early intervention that supports and inspires parents in their parenthood with the purpose of creating favorable conditions for the positive development of their children [2] and encouraging fathers to be involved [28, 30, 31]. It is important to note that the life conditions for the interviewed fathers included several adversities and challenges in the disadvantaged neighborhood that required an ability to adapt to these realities.

Related to evidence-based strategies to reduce health inequalities [40], the extended home visiting program might contribute by providing a good start for life through parental support and improved paternal confidence for the migrant men in their new role as parents. It further facilitates access to health care and other services in society, that are known to increase health literacy (HL) and can lead to strengthened resilience in the study population [41].

### Limitations

Seven of the nine interviews for this study were not conducted in the primary language of the participants, which could limit the fathers’ ability to express themselves freely. Further six interviews with the participants were conducted at their homes, while their wife and child were at home, which may have affected the participants’ opportunity to express themselves freely. This study was explorative and focused on fathers´ experiences in their life situation including their experiences of the home visiting program. As a small qualitative study its findings were not meant to be generalised. The findings of this study presented experiences from the migrant fathers’ perspectives and cannot indicate if paternal involvement into this program might increase fathers’ involvement or improve children’s outcomes [33].

## Conclusions

In terms of resilience, the extended postnatal home visiting program benefitted migrant fathers by meeting part of their needs for support on an individual level, both in knowledge and in parental confidence, and on a structural level, as they gained information about available societal services and resources in their local area.

The participating fathers’ descriptions of striving for stability in their living conditions revealed their inherent adaptability (resilience) to their new life situation. Further studies are suggested to explore the ways by which extended home visiting programs can affect parental health literacy levels and possibly strengthen resilience among migrant parents. Further studies regarding migrant fathers’ experiences are warranted.

## Additional file

Additional file 1: Draft of the interview guide - parents who has participated in the extended postnatal home visiting program. (DOCX 15 kb)

## Abbreviations

CHC: Child Health Care
CHCC: Child Health Care Center
GT: Grounded Theory
HL: Health Literacy
