# Supplementary material for: Strengthening resilience through an extended postnatal home visiting program in a multicultural suburb in Sweden: fathers striving for stability
Source: BMC Public Health. 2019 Jan 22;19:102. doi: 10.1186/s12889-019-6440-y (PMC6343238; doi:10.1186/s12889-019-6440-y)
Supplement: Supplementary file 1 — Draft of the interview guide - parents who has participated in the extended postnatal home visiting program. (DOCX 15 kb) [file 12889_2019_6440_MOESM1_ESM.docx]

13^th^ of May 2013

**Draft of the interview guide - parents who has participated in the extended postnatal home visiting program**

**Health and well-being**

- How would you describe your health? Do you have any problems with your health? How your health is now compared to when your child was just a couple of months old? (since the time of the first interview)
- How is your general life situation? Are you still living with your spouse/partner?
- How would you describe your financial and housing situation?
- How do you perceive your social contacts, do you spend time with family or friends? How do you meet? How do you keep in touch? In which way are they important for you?

**The child**

- Can you describe an ordinary day of your life with your child? What are you used to do?
- How is your child doing? Has your child been healthy? Are you worried about the development of your child?
- How would you describe your child?
- How do you perceive your contact with your child? Do you feel you can comfort your child?
- What is the most difficult thing with your child? What is the nicest thing with your child?
- Breastfeeding, ow did it go? Can you tell me about your experiences?
- Do you visit the open preschool? Do you visit other places or activities that are for children and parents? Which ones? Can you give some examples of what you use to do?

**The extended home visitng program**

- How has the contact with the Child Health Care (CHC) been? You have got home visits by a CHC nurse and a parental advisor. What are your experiences of the home visits? Can you describe what happened during the visits? In which ways were the home visits good or bad? Would it be enough to just visit CHC? How was the parental advisor contributing? How did the CHC contribute? Have the number of visits/meetings with the CHC nurse/the parental advisor been enough?
- You discussed for example feeding, accidents and child development, what do you think about the themes that you discussed during the visits?
- Would you prefer something else to discuss when you met the CHC nurse and the parental advisor? If yes, what?
- Do you think it is a good way (home visiting) to work with families that have their first child? Why?
- Would you like any other kind of support or help regarding the development of your child or your own health or life situation? If yes, what?

**Future**

- What do you think about the future? What do you think is most important for your child? And for you? What are you planning to do in the near future? To work or study and/or have several children, live in this neighborhood or move?
- What do you think about daycare? (What is good and what is not so good?)
- Do you plan for your child to start going to daycare? When?
- Would you like to add something?
